# The Majority of United States Citizens With Cancer do not Have Access to Carbon Ion Radiotherapy

**DOI:** 10.3389/fonc.2022.954747

**Published:** 2022-07-08

**Authors:** Robert L. Foote, Hirohiko Tsujii, Reiko Imai, Hiroshi Tsuji, Eugen B. Hug, Tatsuaki Kanai, Jiade J. Lu, Juergen Debus, Rita Engenhart-Cabillic, Anita Mahajan

**Affiliations:** ^1^ Department of Radiation Oncology, Mayo Clinic College of Medicine and Science, Rochester, MN, United States; ^2^ QST Hospital, Chiba, Japan; ^3^ Department of Bone and Soft Tissue Tumors, QST Hospital, Chiba, Japan; ^4^ International Particle Therapy Research Center Director, QST Hospital, Chiba, Japan; ^5^ MedAustron Ion Therapy Center, Wiener Neustadt, Austria; ^6^ Department of Radiation Oncology and Radiation Therapy, Osaka Heavy Ion Therapy Center, Osaka, Japan; ^7^ Department of Radiation Oncology, Shanghai Proton and Heavy Ion Center, Shanghai, China; ^8^ Department of Radiation Oncology and Radiation Therapy, Heidelberg Ion Beam Therapy Center, Heidelberg, Germany; ^9^ Marburg Ion Beam Radiation Center, University Hospital Marburg, Marburg, Germany

**Keywords:** radiation therapy, carbon ion radiotherapy, US citizens, clinical trial accrual, heavy particle therapy, access

## Abstract

As of December 31, 2020, there were 12 facilities located in Asia and Europe which were treating cancer patients with carbon ion radiotherapy (CIRT). Between June 1994 and December 2020, 37,548 patients were treated with CIRT worldwide. Fifteen of these patients were United States (U.S.) citizens. Using the Surveillance, Epidemiology, and End Results cancer statistics database, the Mayo Clinic in Rochester, MN has conservatively estimated that there are approximately 44,340 people diagnosed each year in the U.S. with malignancies that would benefit from treatment with CIRT. The absence of CIRT facilities in the U.S. not only limits access to CIRT for cancer care but also prevents inclusion of U.S. citizens in phase III clinical trials that will determine the comparative effectiveness and cost effectiveness of CIRT for a variety of malignancies for FDA approval and insurance coverage. Past and present phase III clinical trials have not been able to enroll U.S. citizens due to their unwillingness or inability to travel abroad for CIRT for an extended period. These barriers could be overcome with a limited number of CIRT facilities in the U.S.

## Introduction

Carbon ion radiotherapy (CIRT) is a more physically targeted form of radiotherapy compared to photon (X-ray) or proton radiotherapy due to a sharper lateral beam penumbra and end of range Bragg Peak (1–4). One of the advantages of this more targeted therapy is a lower risk for radiation-induced malignancies (5). It also has the biologic advantages of high linear energy transfer (LET), low oxygen enhancement ratio (OER) and increased relative biological effectiveness (RBE) resulting in more irreparable, clustered DNA double strand breaks (1–4).

A series of dose-escalating phase I/II and phase II clinical trials conducted at QST Hospital (National Institutes for Quantum and Radiological Science and Technology) in Chiba, Japan since 1994 have demonstrated that CIRT is safe and effective in the treatment of a number of recurrent, previously irradiated, photon-based radiation-resistant, and/or hypoxic malignancies including prostate cancer, salivary gland cancer, bone and soft tissue sarcoma, paranasal sinus mucosal melanoma, recurrent rectal cancer, non-small cell lung cancer, and liver cancer (6). These initial findings are being confirmed in Europe (7). Phase III clinical trials are underway in Asia and Europe (clinical trials.gov: NCT04592861, NCT02838602, NCT01182779, NCT01182753, NCT02986516, NCT04536649), yet CIRT is currently not available in the United States (U.S.).

This original evaluation was conducted to determine the number of U.S. citizens who have traveled abroad to receive CIRT. In addition, we present a case series describing the types of malignancies which have been treated in U.S. patients, and patient outcomes including survival, tumor control, and toxicity.

## Methods and Materials

The Particle Therapy Co-Operative Group (PTCOG) website (www.ptcog.ch) was used to identify all CIRT facilities in the world treating patients as of December 31, 2020. The CIRT facilities were contacted to determine if they have treated U.S. citizens who have traveled abroad for CIRT from the date their facility opened and treated their first patient through December 31, 2020. U.S. citizens who had traveled abroad were verified by passport information. U.S. citizens living abroad and non-U.S. citizens living within the U.S. were not included.

The facilities having treated U.S. citizens were invited to provide demographic and outcomes information for their patients according to the policies and regulations of their local institutional review board or ethics committee including informed consent when required. No patient identifying information was shared. A data use agreement was instituted between the participating institutions. The data shared are shown in **Table 1**.

**Table 1:** Patient demographics, diagnosis, staging, treatment, and outcomes.

Patient number	1	2	3	4	5
Date of Diagnosis	10/28/2016	9/25/2017	10/17/2017	7/27/2018	4/1/2020
Age at diagnosis	53	62	52	53	41
Date of death	Alive	Alive	Alive	Alive	Alive
Gender	Female	Male	Male	Male	Male
Diagnosis	Sacral sarcoma	Soft palate cancer	Prostate cancer	Prostate cancer	Sacral chordoma
Pathology	Spindle cell rhabdomyosarcoma	Adenoid cystic carcinoma	Adenocarcinoma (Gleason score 3 + 3 = 6)	Adenocarcinoma (Gleason score 4 + 3 = 7)	Chordoma
Stage UICC	cT2bN0M0	cT3N0M0	cT1cN0M0	cT1cN0M0	NA
Stage AJCC 8^th^ edition	cT4aN0M0	cT3N0M0	cT1cN0M0	cT1cN0m0	NA
Prior treatment	Chemotherapy^	None	Androgen deprivation therapy, LHRH agonist, 2 months	None (patient declined androgen deprivation therapy)	None
ECOG/Zubrod Performance Status	1	0	0	0	0
Date of CIRT treatment	2/3-3/2/2017	2/16-3/11/2018	9/18-10/15/2018	10/26-11/15/2018	7/30-8/26/2020
Total dose, Gy (RBE 1.0)	70.4*	64.0*	51.6*	51.6*	73.6
Dose per fraction, Gy (RBE1.0)	4.4	4.0	4.3	4.3	4.6
Number of fractions	16	16	12	12	16
Fractions per week	4	4	4	4	4
Overall treatment time (days)	28	24	28	21	28
RBE/Dose calculation model used	MKM model	MKM model	MKM model	MKM model	LEM I/pencil beam
Single target volume or small boost volume too	Single target volume	Single target volume	Smaller boost volume included^†^	Smaller boost volume included^†^	Smaller boost volume included-GTV^‡^
Carbon ions only or mixed beam (photons or protons)	Carbon ions only	Carbon ions only	Carbon ions only	Carbon ions only	Carbon ions only
Use of concurrent hormonal therapy	None	None	None	None	None
Use of concurrent chemotherapy or other medical therapy	None	None	None	None	None
Baseline pretreatment signs and symptoms related to the current cancer using CTCAEv5	Grade 2 walking disability Grade 2 peripheral nerve (sciatic nerve) Functional mobility scale 4 (requires a cane)	Soft palate swelling	None	Grade 1 urinary frequency	Grade 3 tumor pain Functional mobility scale 4 (requires a cane) Grade 2 urinary incontinence Grade 3 erectile dysfunction
Acute adverse events, CTCAEv5					
During treatment	Grade 2 walking disability Grade 2 peripheral nerve (sciatic nerve) Functional mobility scale 4 (requires a cane)	Grade 2 oral mucosa Grade 1 skin	None	Grade 1 genitourinary	None
90 days following treatment	N/A	N/A	N/A	N/A	N/A
Late adverse events, CTCAEv5					
>90 days following treatment	Grade 2 walking disability Grade 2 peripheral nerve (sciatic nerve) Functional mobility scale 4 (requires a cane)	N/A	N/A	None (Grade 0 gastrointestinal and genitourinary)	None
Tumor response as determined by clinical evaluation and imaging	Metabolic complete response by PET. Stable disease by size. Imaging June 2020	N/A	PSA 1.37 and fPSA 0.094 on June 5, 2020 Asymptomatic clinically on 9/4/2020	No evidence of disease on 5/16/2019. Asymptomatic clinically on 8/29/2020	Partial response, 48% volume reduction by MRI (1178 cc to 606 cc), Grade 0 tumor pain Grade 0 urinary incontinence Functional mobility scale 6 (independent on all surfaces, no walking aids needed) Grade 1 erectile dysfunction
Patient survival status	Alive, no evidence of disease on 10/11/2020	Alive, no evidence of disease on 7/31/2020	Alive, no evidence of disease on 9/4/2020	Alive, no evidence of disease on 8/29/2020	Alive, 8/30/2021

^Docetaxel, gemcitabine, and doxorubicin- 2 courses.

*RBE-weighted dose approximated to a median dose in the planning target volume.

^†^For treatment planning, the clinical target volume (CTV) was defined as the whole prostate and the proximal one-third or half of the seminal vesicles (SV) for T1-T3a disease. The planning target volume (PTV)1 was defined as the CTV plus 5-mm margins in the cranial, caudal and posterior directions and 10-mm margins in the right, left and anterior directions. PTV1 was used for the first 8 treatments. The PTV2 was created by adding 2-3 mm margins from the dorsal aspect of the CTV and was identical to the CTV in the cranial and caudal directions; PTV2 was used for the last 4 treatments of the whole treatment course.

^‡^PTV1 received 9 fractions for a total dose of 41.4 Gy (RBE1.0). A sequential boost to PTV2 included an additional 7 fractions for a total dose of 73.6 Gy (RBE1.0) (41.4 Gy [RBE1.0] plus 32.2 Gy [RBE1.0]). CTV1 was defined as gross tumor volume (GTV) and the piriform muscles bilaterally plus a margin > 1cm in gluteal muscle and sacral bone up to the sacroiliac joints. CTV2 was defined as GTV plus 1 cm adapted to anatomy. Two patient treatment positions were used, prone and lateral decubitus, with a fixed horizontal beam.

NA, not applicable; LHRH, luteinizing hormone-releasing hormone; MKM, Microdosimetric Kinetic Model; LEM, Local Effect Model; GTV, gross tumor volume; N/A, not available.

## Results

As of December 31, 2020, there were 12 facilities located in Asia and Europe which were treating cancer patients with CIRT (**Table 2**). Four facilities have treated U.S. citizens. Two facilities agreed to provide patient data. Between June 1994 and December 2020, 37,548 patients were treated with CIRT worldwide (**Table 2**). Fifteen of these patients were U.S. citizens (**Table 2**).

**Table 2:** Number of U.S. Citizens Treated with CIRT.

Facility	Location	First treatments (year)	Total Patients Treated*	U.S. Citizens Treated	Self-referred	Physician referred
MedAustron	Austria	2019	185 (8/2021)	1^		1
Shanghai Proton and Heavy Ion Center	China	2014	3141 (9/2021)	0^†^		
Heavy Ion Cancer Treatment Center-Wuwei Cancer Hospital, Gansu Province	China	2020	400 (8/2021)	0		
Heidelberg Ion Beam Therapy Center	Germany	2009/2012	3468 (12/2019)	7		
Marburg Ion Beam Therapy Center	Germany	2015^‡^	430 (12/2019)	3		
CNAO-Pavia	Italy	2012	1815	0		
HIMAC, QST Hospital, Chiba	Japan	1994/2017	13,489 (12/2019)	4	4	
HIBMC-Hyogo	Japan	2002	3119	0		
GHMC-Gunma	Japan	2010	4560	0		
SAGA-HIMAT-Tosu	Japan	2013	5000 (8/2020)	0		
i-Rock Kanagawa Cancer Center	Japan	2015	1541	0		
Osaka Heavy Ion Therapy Center	Japan	2018	400 (12/2019)	0		
Total			37,548	15		

*As of December 2020, unless otherwise indicated.

^Two patients from Israel underwent surgery in the U.S. and were subsequently referred by their U.S. physician to MedAustron for CIRT. Both had malignancies involving the skull base. One was a primary previously untreated case and the other was recurrent after prior radiotherapy.

^†^Expatriates living in the U.S. have been treated in Shanghai, China.

^‡^The Marburg Ion Beam Therapy Center initially treated patients from 2015 through 2018 under the direction of Prof. Juergen Debus from the University of Heidelberg. The University Radiation Clinic Marburg became responsible for operating the Marburg Ion Beam Radiation Center (MIT) under the direction of Prof. Dr. med. Rita Engenhart-Cabillic in January 2019.

CNAO, Centro Nazionale di Adroterapia Oncologica (National Center of Oncological Hadrontherapy); HIMAC, Heavy Ion Medical Accelerator in Chiba; HIBMC, Hyogo Ion Beam Medical Center; GHMC, Gunma University Heavy Ion Medical Center; SAGA-HIMAT, Saga Heavy Ion Medical Accelerator in Tosu; i-ROCK, Ion-beam Radiation Oncology Center in Kanagawa.

In addition, 6 facilities are under construction in China, France, Japan, South Korea (2), and Taiwan.

Two facilities have closed and are no longer treating patients, Lanzhou, China, 2006-2013, 213 patients treated; Gesellschaft für Schwerionenforschung (GSI, Institute for Heavy Ion Research) in Darmstadt, Germany, 1997-2009, 440 patients treated.

Demographic, diagnostic, pathologic, staging, treatment, toxicity, tumor response, and survival data were made available for 5 patients (**Table 1**). The median age was 53 years (41-62 years). 4 patients were male. Two patients had prostate cancer, one patient had adenoid cystic carcinoma of the soft palate, one patient had a rhabdomyosarcoma of the sacrum and one patient had a sacral chordoma. All patients were ECOG or Zubrod performance status 0 or 1. There was no grade 3 or higher acute or late toxicity associated with CIRT. All patients remain alive with no evidence of disease 12 to 43 months (median 24 months) since completing CIRT.

## Discussion

After a thorough evaluation, to the best of our knowledge, 15 U.S. citizens have traveled abroad to receive CIRT. Using the Surveillance, Epidemiology, and End Results cancer statistics database, the Mayo Clinic in Rochester, MN has conservatively estimated that there are approximately 44,340 people diagnosed each year in the U.S. with malignancies that would benefit from treatment with CIRT. Approximately 780 patients with an indication for treatment with CIRT undergo evaluation at Mayo Clinic in Rochester, MN each year.

Despite this need, no CIRT facilities exist within the U.S. and barriers limit access to international facilities. Not all CIRT facilities accept international patients. Third-party medical coordinators are available to provide contracted services to patients seeking CIRT including review of medical records to determine eligibility for CIRT and arranging for international travel, housing, in-country transportation, insurance coverage and translation services as needed. Nevertheless, these remain substantial barriers to U.S. citizens seeking CIRT abroad. U.S. citizens face some of these same challenges even when seeking tertiary and quaternary medical care for complex illnesses within the U.S. with preference given to health care closer to home (8, 9), and insurance limitations on where they can receive care and which treatments are covered. Not all physicians or patients are aware of CIRT which may further limit referral. Referral outside of the provider’s network and associated revenue concerns may also be hurdles to referring patients for CIRT.

Additional barriers could include patient’s performance status and time away from family, friends, and work. A typical course of CIRT would require patients travel abroad for up to six weeks to complete a typical course of treatment of 4 days per week for 4 weeks with another week or two for treatment planning time. The global COVID-19 pandemic has introduced additional anxiety and barriers to travel over he past two years which could impact future international mobility.

Telemedicine has the potential to mitigate some, but not all, of the above barriers. Videoconferencing could be used to provide consultations to determine if the patient is eligible for CIRT prior to traveling abroad. This platform could also be used to provide patient education about the complexity of CIRT, introduce and engage the patient with their CIRT care team, and provide at home follow up care coordinated between the referring physician and the foreign CIRT care team limiting the number of trips and time abroad to the treatment planning and delivery time. Clinical trial information could also be obtained by electronic means.

Lazar, et al. have reported an increase in the number of trials investigating CIRT since 2010, and the number of countries and sites offering CIRT is slowly growing. They note, however, this progress has excluded other countries, including the U.S. They proposed several recommendations to study CIRT to accelerate progress in the field, including: 1) increasing the number of multinational randomized clinical trials, 2) leveraging the existing CIRT facilities to launch larger multinational trials directed at common cancers combined with high-level quality assurance; and 3) developing more compact and less expensive next-generation treatment systems integrated with radiobiologic research and preclinical testing (10). As noted above, barriers exist to U.S. citizens which limit benefits from successful implementation of recommendation 1 and 2. Implementation of recommendation 3 has the potential to improve the feasibility of construction of a limited number of CIRT facilities in the U.S. which would reduce many of the barriers.

The absence of U.S. CIRT facilities not only limits access to CIRT for cancer care but also prevents inclusion of U.S. citizens in phase III clinical trials that will determine the comparative effectiveness and cost effectiveness of CIRT for a variety of malignancies for FDA approval and insurance coverage. Past and present phase III clinical trials have not been able to enroll U.S. citizens due to their unwillingness or inability to travel abroad for an extended period due to the barriers noted above. These barriers could be overcome with a limited number of CIRT facilities in the U.S. with perhaps one located in the Eastern, Midwestern, and Western U.S.

The ideal CIRT facilities in the U.S. would have existing patient volumes to match the capacity of the facility, allow easy and rapid access to CIRT *via* responsive, respected radiation oncologists with particle therapy experience and subspecialty expertise in malignancies with indications for CIRT who will communicate openly and in a timely manner with referring physicians, patients and their families; radiation therapists, certified medical dosimetrists and medical physicists with particle therapy expertise; educational infrastructure to train the next generation of CIRT providers, research infrastructure to conduct basic science and clinical research, and a comprehensive cancer and medical center to provide surgery, medical therapy, rehabilitative services, social services, psychologic support, chaplain services and spiritual support, and concierge services to help patients and family members with transportation, food and housing, entertainment, and insurance coverage.

## Conclusion

Fifteen U.S. citizens have been treated with CIRT. Substantial barriers to traveling abroad for CIRT exist. A limited number of CIRT facilities in the U.S. would improve access to CIRT and enhance accrual to phase III clinical trials. U.S. patients deserve more practical access to life saving cancer treatments which are available elsewhere in the world.

## Data Availability Statement

The raw data supporting the conclusions of this article will be made available by the authors, without undue reservation.

## Ethics Statement

This study was reviewed and approved by QST Hospital. Written informed consent for participation was not required for this study in accordance with national legislation in the United States and institutional requirements of Mayo Clinic.

## Author Contributions

All authors have made substantial contributions to conception and design, acquisition of data, analysis, and interpretation of data; and have been involved in drafting the manuscript or revising it critically for important intellectual content; and given final approval of the version to be published. Each author has participated sufficiently in the work to take public responsibility for appropriate portions of the content; and agreed to be accountable for all aspects of the work in ensuring that questions related to the accuracy or integrity of any part of the work are appropriately investigated and resolved.

## Conflict of Interest

The authors declare that the research was conducted in the absence of any commercial or financial relationships that could be construed as a potential conflict of interest.

## Publisher’s Note

All claims expressed in this article are solely those of the authors and do not necessarily represent those of their affiliated organizations, or those of the publisher, the editors and the reviewers. Any product that may be evaluated in this article, or claim that may be made by its manufacturer, is not guaranteed or endorsed by the publisher.
